# Molecular and Serological Characterization of Hepatitis B Virus (HBV)-Positive Samples with Very Low or Undetectable Levels of HBV Surface Antigen

**DOI:** 10.3390/v13102053

**Published:** 2021-10-13

**Authors:** Mary C. Kuhns, Vera Holzmayer, Mark Anderson, Anne L. McNamara, Silvia Sauleda, Dora Mbanya, Pham T. Duong, Nguyen T. T. Dung, Gavin A. Cloherty

**Affiliations:** 1 Abbott Laboratories, Infectious Diseases Research, Diagnostics Division, 100 Abbott Park Road, Abbott Park, IL 60064, USA; Vera.Holzmayer@abbott.com (V.H.); Mark.Anderson6@abbott.com (M.A.); almcnamara4049@gmail.com (A.L.M.); Gavin.Cloherty@abbott.com (G.A.C.); 2 Laboratori de Seguretat Transfusional BST, Banc de Sang I Teixits Passeig Taulat, 08005 Barcelona, Spain; ssauleda@bst.cat; 3 Department of Hematology, University of Yaounde, Yaounde BP 8046, Cameroon; dmbanya1@yahoo.co.uk; 4 Ministry of Health-National Institute of Hematology and Blood Transfusion, Pham Van Bach St., Yen Hoa, Cau Giay, Ha Noi 122000, Vietnam; pduongmd@gmail.com (P.T.D.); thanhdungnihbt@gmail.com (N.T.T.D.)

**Keywords:** hepatitis B virus, hepatitis B surface antigen, occult hepatitis B, HBV biomarkers

## Abstract

Background: Gaps remain in the detection of nucleic acid test (NAT) yield and occult hepatitis B virus (HBV) infection (OBI) by current HBV surface antigen (HBsAg) assays. The lack of detection may be due to HBsAg levels below current assay detection limits, mutations affecting HBsAg assays or HBsAg levels, or the masking of HBsAg by antibody to HBsAg (anti-HBs). In this study, we evaluate the incremental detection of NAT yield and OBI from five diverse geographic areas by an improved sensitivity HBsAg assay and characterize the samples relative to the viral load, anti-HBs status, and PreS1–S2–S mutations. Included is a comparison population with HBV DNA levels comparable to OBI, but with readily detectable HBsAg (High Surface–Low DNA, HSLD). Methods: A total of 347 samples collected from the USA, South Africa, Spain, Cameroon, Vietnam, and Cote D’Ivoire representing NAT yield (HBsAg(−), antibody to HBV core antigen (anti-HBc)(−), HBV DNA(+), N = 131), OBI (HBsAg(−), anti-HBc(+), HBV DNA(+), N = 188), and HSLD (HBsAg(+), anti-HBc(+), HBV DNA(+), N = 28) were tested with ARCHITECT HBsAg NEXT (HBsAgNx) (sensitivity 0.005 IU/mL). The sequencing of the PreS1–S2–S genes from a subset of 177 samples was performed to determine the genotype and assess amino acid variability, particularly in anti-HBs(+) samples. Results: HBsAgNx detected 44/131 (33.6%) NAT yield and 42/188 (22.3%) OBI samples. Mean HBV DNA levels for NAT yield and OBI samples were lower in HBsAgNx(−) (50.3 and 25.9 IU/mL) than in HBsAgNx(+) samples (384.1 and 139.5 IU/mL). Anti-HBs ≥ 10 mIU/mL was present in 28.6% HBsAgNx(+) and 45.2% HBsAgNx(−) OBI, and in 3.6% HSLD samples. The genotypes were A1, A2, B, C, D, E, F, and H. There was no significant difference between HBsAgNx(−) and HBsAgNx(+) in the proportion of samples harboring substitutions or in the mean number of substitutions per sample in PreS1, PreS2, or S for the NAT yield or OBI (*p* range: 0.1231 to >0.9999). A total of 21/27 (77.8%) of HBsAgNx(+) OBI carried S escape mutations, insertions, or stop codons. HSLD had more PreS1 and fewer S substitutions compared to both HBsAgNx(−) and HBsAgNx(+) OBI. Mutations/deletions associated with impaired HBsAg secretion were observed in the OBI group. Conclusions: HBsAgNx provides the improved detection of NAT yield and OBI samples. Samples that remain undetected by HBsAgNx have exceptionally low HBsAg levels below the assay detection limit, likely due to low viremia or the suppression of HBsAg expression by host and viral factors.

## 1. Introduction

More than 257 million people are chronically infected with the hepatitis B virus (HBV) globally [1]. The hepatitis B surface antigen (HBsAg) remains the foundation for the diagnosis of acute and chronic infection, for screening blood and blood products, and for defining the cure in response to antiviral therapy. A hallmark of the hepatitis B virus infection is the presence of a large excess of subviral particles containing HBsAg but lacking hepatitis B viral DNA (HBV DNA). The magnitude of the excess of HBsAg over HBV DNA-containing virions can vary widely depending on the phase of infection [2,3,4,5,6]. During the early phase of acute infection, HBV DNA may be the only detectable marker. In occult HBV infection (OBI), HBV DNA is present in the liver or plasma, but serum HBsAg is undetectable by currently available assays; antibody to HBV core antigen (anti-HBc) and/or antibody to HBsAg (anti-HBs) may be present or absent. An improved HBsAg detection is of particular importance in the diagnosis of early acute and OBI [5,7]. A number of factors can affect the ability of immunoassays to detect HBsAg, including analytical sensitivity, the possible concealment of HBsAg by anti-HBs, HBsAg mutations that may decrease the binding of antibodies used in HBsAg assays, and HBV mutations that reduce HBsAg production and/or secretion to levels below the detection limits of current HBsAg assays [5,8].

We recently reported on the performance of a new, highly sensitive HBsAg assay (ARCHITECT HBsAg NEXT) with an analytical sensitivity of 0.005 IU/mL, a specificity of 99.95–100% in blood donor and diagnostic populations, and the increased detection of early acute and occult hepatitis B infections [9,10,11,12]. The present study significantly expands on our earlier work to evaluate incremental detection by the new HBsAg assay in a larger sample population representing early acute and OBI from five geographic regions and diverse genotypes. We explore factors that may affect the detection of low levels of HBsAg by performing extensive serologic and molecular analyses, including the sequencing of the PreS1, PreS2, and S gene regions of HBsAg NEXT-negative and HBsAg NEXT-positive samples. For comparison, we included a sample population representing another phase of the HBV serological spectrum with low HBV DNA levels (comparable to early acute and OBI), but having readily detectable levels of HBsAg. In addition, we assess the potential value of two new biomarkers, HBV pregenomic RNA (pgRNA) and the hepatitis B core-related antigen (HBcrAg), in detection of OBI. The evidence indicates that the early acute and OBI samples that remain undetected by HBsAg NEXT have extremely low levels of HBsAg below the assay detection limit.

## 2. Materials and Methods

### 2.1. Samples

A total of 319 blood donor samples collected from the USA (N = 95), South Africa (N = 84), Spain (N = 30), Cameroon (N = 21), and Vietnam (N = 89) representing early acute (identified as HBV nucleic acid test (NAT) yield) and OBI were evaluated. In addition, 28 samples with the serological pattern of high HBsAg with low HBV DNA (HSLD) were included for comparison. Countries of origin for the HSLD samples were Cameroon (N = 1), USA (N = 3), Vietnam (N = 5), and Cote D’Ivoire (N = 19). HBV NAT yields (N = 131) were ARCHITECT HBsAg Qualitative II-negative, anti-HBc-negative, and HBV DNA-positive. OBI samples (N = 188) were ARCHITECT HBsAg Qualitative II-negative, anti-HBc-positive, and HBV DNA-positive. Eighty-one (43.1%) of OBI samples were antibody to hepatitis B e antigen (anti-HBe)-positive. HSLD (N = 28) samples were anti-HBc-positive and HBsAg-positive, anti-HBc IgM-negative, hepatitis B e antigen (HBeAg)-negative, with HBV DNA levels comparable to the OBI population. Twenty-six (92.9%) of HSLD samples were anti-HBe-positive. NAT yield and OBI samples were provided by Banc de Sang I Teixits, Spain; Universite des Montagres, Cameroon; Ha Noi National Institution of Hematology and Blood Transfusion, Vietnam; purchased from the American Red Cross (Gaithersburg, MD, USA), the South African National Blood Service (Boksburg, South Africa), or Boca Biolistics (Pompano Beach, FL, USA). HSLD samples from blood donors or untreated diagnostic populations were purchased from ProMedDx (Norton, MA, USA) and Boca Biolistics (Pompano Beach, FL, USA). All samples were from untreated HBV-infected individuals. No samples were from patients co-infected with HCV. Nine samples from Cameroon were co-infected with HIV. In this study, samples were considered HBV DNA-positive if at least two determinations were positive using Abbott RealTi*m*e HBV, Procleix Ultrio Discriminatory Assay for HBV (Grifols, Emeryville, CA, USA), Cobas MPX (Roche, Indianapolis, IN, USA), or PCR of the preS-S region (method described below).

### 2.2. Serologic and Molecular Assays

Samples were tested for HBsAg using ARCHITECT HBsAg Qualitative II (analytical sensitivity 0.017–0.022 IU/mL, Abbott, Sligo, Ireland) and with the new qualitative ARCHITECT HBsAg NEXT (HBsAgNx) assay with analytical sensitivity of 0.005 IU/mL [9,12]. HBsAgNx is a one-step chemiluminescent microparticle immunoassay with two monoclonal antibodies coated on the microparticles and a goat anti-HBs conjugate. The assay uses 75 µL of specimen (the same as ARCHITECT Qualitative II) with no sample pretreatment. The assay is fully automated and was performed on the Abbott ARCHITECT or Alinity *i* instruments equipped with heat induction probes to eliminate sample carry-over. Ancillary wash buffer was added in a second incubation step so the instrument performed a two-step assay protocol. Samples repeatedly reactive by HBsAgNx were confirmed by neutralization with sheep anti-HBs in the new ARCHITECT HBsAg NEXT Confirmatory assay (Abbott, Sligo, Ireland). Anti-HBc, quantitative anti-HBs, quantitative HBsAg, and anti-HBe assays were performed using ARCHITECT assays (Abbott, Sligo, Ireland or Wiesbaden, Germany). HBV DNA levels were quantitated with Abbott RealTi*m*e HBV (Abbott Molecular, Des Plaines, IL, USA). Results of the RealTi*m*e HBV assay were expressed as target not detected, detected below the lower limit of quantitation (i.e., HBV DNA detected but not quantifiable), or a calculated result in IU/mL if within the assay linear range. The lower limit of quantitation was 10 IU/mL (34.1 HBV DNA copies/mL) for the 0.5 mL sample preparation protocol. The assay was standardized using the WHO International Standard. Samples with detectable HBV DNA below the quantitation range were assigned the value of 0.1 log IU/mL for the purposes of analysis in this study. Hepatitis B core-related antigen (HBcrAg) testing was performed with the LUMIPULSE G HBcrAg assay (Fujirebio, Tokyo, Japan) with a lower limit of measurement of 3 log U/mL. HBV pregenomic RNA (HBV pgRNA) was determined using a research assay with a lower limit of quantitation of 1.65 log U/mL as previously described [13]. Results for samples with detectable pgRNA below the quantitation range were reported as <1.65 log U/mL.

### 2.3. Sequencing of the PreS1–PreS2–S Gene Regions

HBV DNA was extracted from 0.5 mL of plasma using the automated protocol DNA-protK-500-50 (research use only) on the *m*2000*sp* system (Abbott Molecular, Des Plaines, IL, USA). First- and second-round PCR were performed to amplify the preS1–S region using AmpliTaq Gold^®^ DNA polymerase (Applied Biosystems, Foster City, CA, USA). First-round primers were HBV2813 F (5′- TCATTTTGTGGGTCACCATATT-3′, nt 2811–2832) and 18R (5′−CCCATGAAGTTTAGGGAATAAC-3′, nt 860–881); second-round primers were HBV-2822 F (5′- GGGTCACCAT ATTCTTGGGAAC-3′, nt 2820–2841) and 19R (5′- GTTAGGGTTTAAATGTATACCC-3′, nt 822–843), amplifying a 1245 base pair fragment. The 50 µL PCR reaction (first and second rounds) contained 0.4 µM of each primer, 2.5 mM MgCl_2_, 0.8 µM dNTP mix and 25 µL extracted DNA for first-round PCR, or 2 µL of first-round PCR as template for the second round. First- and second-round amplifications consisted of preincubation at 95 °C (10 min), 40 cycles at 95 °C (20 s), annealing at 50 °C (45 s), extension at 66 °C (2.5 min for the first-round or 1.5 min for the second round), and final extension at 72 °C (10 min). Both strands of purified amplification products were sequenced directly using the BigDye^®^ Terminator v3.1 Cycle Sequencing kit and the ABI 3130*xl* Genetic Analyzer (Applied Biosystems, Foster City, CA, USA). Sequence data were assembled and edited using Sequencher software (v5.4.6; Gene Codes Corporation, Ann Arbor, MI, USA). Positions with sequence ambiguities were assigned the appropriate IUPAC designation. Genotype was determined by phylogenetic analysis using the PHYLIP v3.5c software package (J. Felsenstein, University of Washington, Seattle, DC, USA). Nucleotide sequences were aligned with the reference sequences representing genotypes A–I using BioEdit 7.0.4.1 [14].

### 2.4. Mutation Analysis

Atypical amino acid substitutions were determined by comparing the specimen sequences to the genotype consensus sequence created in BioEdit from alignment of genotype sequences downloaded from https://hbvdb.ibcp.fr (accessed on 2 March 2019) [15]. Atypical amino acid substitutions were determined by comparing the specimen sequence to a genotype consensus sequence and defined as amino acids different from consensus with frequencies ≤ 10% in a genotype alignment downloaded from the HBV database. In case of a mixture of wild-type and atypical amino acids, a percentage substitution was estimated proportional to the heights of corresponding chromatograms in a mixed nucleotide call. Mixtures of mutant and wild-type sequences were included in the analyses if the mutation comprised ≥50% of amino acids. Surface gene escape mutations were determined using the Geno-2-pheno-hbv tool (https://hbv.geno2pheno.org/index.php, accessed on 10 July 2021).

### 2.5. Statistical Analysis

Group comparisons were calculated using a two-tailed *t*-test; categorical data were analyzed using Fisher’s exact test. Analyses were performed using GraphPad Prism (8.0.2). Significant *p*-value was <0.05.

## 3. Results

### 3.1. Incremental Detection by ARCHITECT HBsAgNx

Table 1 summarizes serological and molecular data for the three major sample populations in this study (total of 347 samples): blood donor samples identified as the HBV NAT yield (anti-HBc-negative, HBsAg-negative, HBV DNA-positive), OBI (anti-HBc-positive, HBsAg-negative, HBV DNA-positive), and an anti-HBc-positive comparison population with low HBV DNA levels but having HBsAg levels readily detectable by current assays with sensitivity ≥0.05 IU/mL (defined here as High HBsAg–Low HBV DNA, HSLD; mean HBsAg level 10,104 IU/mL). HBsAg levels were ≥174 IU/mL in 25/28 HSLD samples as measured by the quantitative ARCHITECT HBsAg assay. Based on the detection limits of the respective qualitative HBsAg assays, the HBV NAT yield and OBI groups were further stratified relative to their level of HBsAg: <0.005 IU/mL if negative by both HBsAg Qualitative II and HBsAgNx or 0.005–0.02 IU/mL if HBsAg Qualitative II-negative and HBsAgNx-positive. The HBsAgNx assay demonstrated an improved detection of both the NAT yield and OBI populations with an incremental detection of 33.6% for the NAT yield and 22.3% for OBI samples.

### 3.2. HBV DNA and Anti-HBs Levels Relative to HBsAgNx Detection

**Figure 1:**
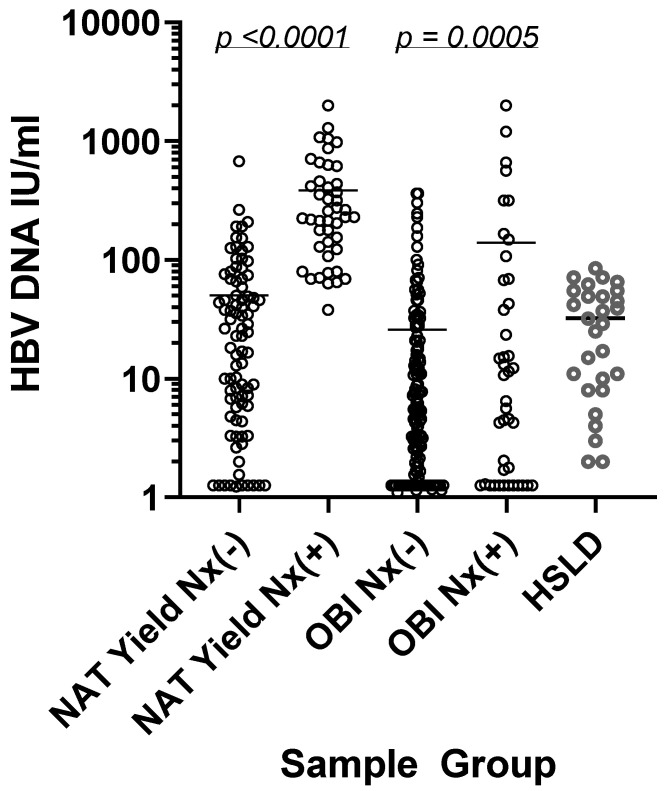
HBV DNA levels in NAT yield, OBI, and HSLD samples: HBsAgNx-negative NAT yield (N = 87), HBsAgNx-positive NAT yield (N = 44), HBsAgNx-negative OBI (N = 146), HBsAgNx-positive OBI (N = 42), HSLD (N = 28). Samples with HBV DNA levels below the quantification range were assigned the value of 0.1 log IU/mL (1.26 IU/mL) for the purpose of analysis. Mean values are indicated by bars.

The mean HBV DNA levels were higher in the NAT yield and OBI samples detected by HBsAgNx (384.1 and 139.5 IU/mL, respectively) than in HBsAgNx negatives (50.3 and 25.9 IU/mL, respectively) (Table 1 and Figure 1). In comparison, the mean HBV DNA level in the HSLD population (32.4 IU/mL) was comparable to the low DNA levels found in the HBsAgNx-negative NAT yield and OBI groups, yet the HSLD group had readily detectable levels of HBsAg.

**Figure 2:**
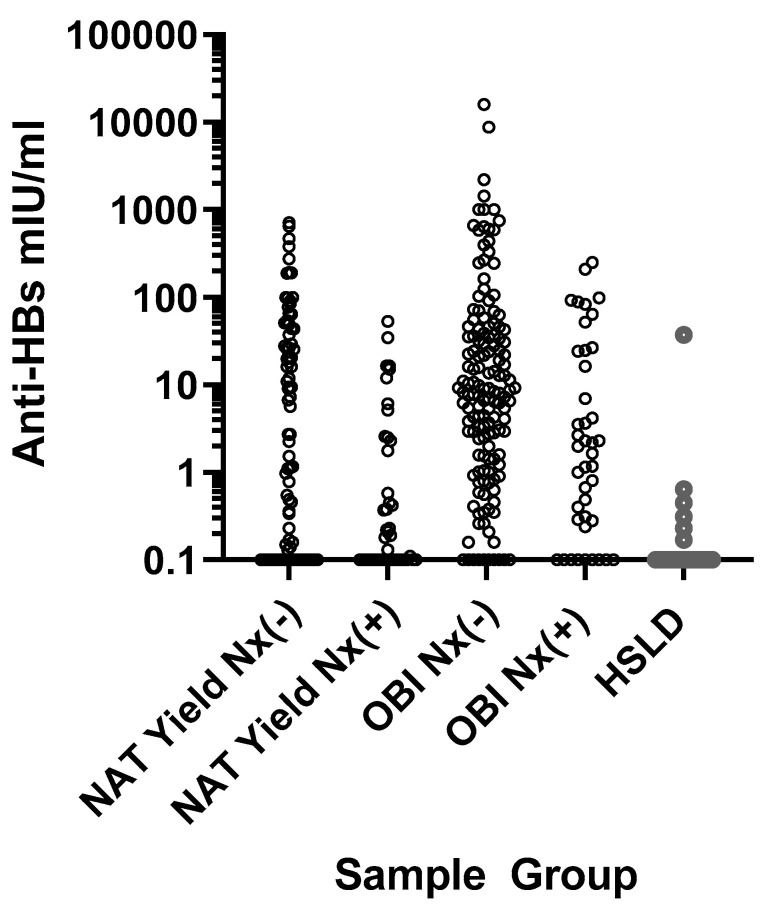
Anti-HBs levels in NAT yield, OBI, and HSLD samples: HBsAgNx-negative NAT yield (N = 87), HBsAgNx-positive NAT yield (N = 44), HBsAgNx-negative OBI (N = 146), HBsAgNx-positive OBI (N = 42), HSLD (N = 28). Values ≥10 mIU/mL were considered positive for anti-HBs.

Positive anti-HBs levels (i.e., ≥10 mIU/mL) were observed in 42 NAT yield samples and 78 OBI samples (Table 1 and Figure 2). Anti-HBs ≥10 mIU/mL was more frequent in the HBsAgNx-negative than HBsAgNx-positive NAT yield samples (*p* = 0.0014), while there was no significant difference for the HBsAgNx-negative vs. HBsAgNx-positive OBI samples (*p* = 0.0748) (Table 1). Only one HSLD sample was anti-HBs-positive. Consistent with our previous observation that the HBsAgNx assay improved the detection of HBsAg in the presence of anti-HBs, 18/86 (20.9%) NAT yield and OBI samples incrementally detected by HBsAgNx were anti-HBs-positive [10,11]. However, a combination of anti-HBs >300 mIU/mL with an extremely low viral load could have limited HBsAg detection (Figure 2). For the 20 samples with anti-HBs >300 mIU/mL, none were HBsAgNx-positive. The median HBV DNA level for these samples was 4.42 IU/mL.

### 3.3. Sequence Analysis of the PreS1, PreS2, and S Regions

The successful amplification and sequencing of the PreS1–PreS2–S region was achieved in 177 out of 213 samples attempted (Table 1). Particular focus was placed on samples with anti-HBs levels ≥10 mIU/mL: 72 of the 213 samples attempted for sequencing had anti-HBs ≥10 mIU/mL and 57 of the 72 were successfully sequenced.

Data were analyzed to evaluate differences in the proportion of samples harboring substitutions and in the mean number of substitutions present in those samples having substitutions. Substitutions were determined by comparison with the consensus sequence for the respective genotype. For the NAT yield, OBI, and HSLD groups, the proportion of samples with substitutions ranged from 53.5% to 92.9% for PreS1, 63.0% to 96.4% for PreS2, and 48.8% to 85.7% for S (Table 1). There was no significant difference in the proportion of samples harboring substitutions or in the mean number of substitutions in PreS1, PreS2, or S between HBsAgNx-negative and HBsAgNx-positive samples for both the NAT yield and OBI groups (*p* values ranged from 0.1231 to >0.9999)

S substitutions classified as escape mutations were identified in 3 NAT yield samples and in 28 OBI samples (Table 1). There was no significant difference in the frequency of S region escape mutations between HBsAgNx-negative and HBsAgNx-positive specimens for the NAT yield population or the OBI population (*p* > 0.9999). None of the samples in this study had the classic sG145R mutation associated with vaccine breakthrough which has been reported at a high prevalence in some studies of OBI (Table 2 and Table 3). One OBI HBsAgNx-positive–anti-HBs-positive sample had a D144N mutation, a rare mutation associated with vaccine breakthrough (Table 3) [10].

Six OBI samples had substitutions at cysteines involved in disulfide bonds within the ‘a’ determinant (amino acids 124–147) (Table 3). None of the NAT yield samples had substitutions at cysteine residues in this region (Table 2). Three samples with C124Y or C124S substitutions were detected by the HBsAgNx assay. Three samples with the C139CY or C147Y/CY substitution were HBsAgNx-negative. The lack of detection was likely due to a low HBsAg concentration since the C139Y and C147Y substitutions have previously been shown to be detectable by HBsAgNx when HBsAg is >0.005 IU/mL [9,12]. All of the ‘a’ determinant substitutions that were observed in HBsAgNx-negative samples in this study were shown to be detectable by HBsAgNx, either from results for other samples in the current study or in previous studies using recombinant and native HBsAg [9,12].

Eight OBI samples had major hydrophilic region (MHR) substitutions associated with an impaired secretion of virions and subviral particles: G119R, Q129R, T140I, and D144A (Table 3) [16]. None of the NAT yield samples carried these substitutions (Table 2). Two HSLD samples and one OBI HBsAgNx-positive sample carried the sW172 stop codon associated with the truncation of surface proteins and impaired viral particle secretion [17]. None of the samples in this study had substitutions at N146 in the S domain, a glycosylation site required for virion secretion [18]. The substitution M133T with either the genotype A1 wild-type N131 or with the substitution T131N in a non-genotype A backbone creates an extra N-linked glycosylation site that reportedly does not affect HBsAg secretion but may mask antigenic sites affecting detection [18]. Two OBI samples had the substitution M133T: one HBsAgNx-negative sample with the genotype A backbone and one HBsAgNx-positive genotype B sample which also carried the substitution T131N (Table 3). Two other OBI samples had substitutions M133T and T131N; however, these were mixed with wild-type sequences which could mitigate the effects of the mutation. Recombinant S protein with the M133T mutation in a genotype A backbone was previously shown to be detectable by HBsAgNx when the concentration is ≥0.005 IU/mL [9].

The highest proportion of samples with substitutions in all three surface antigen regions was observed in the HSLD group (85.7%) (Table 1). In those samples harboring substitutions, there were significantly more PreS1 substitutions and fewer S substitutions in the HSLD group compared to both HBsAgNx-negative and HBsAgNx-positive OBI samples (Table 1). Only two (7.1%) HSLD samples had an S escape mutation compared to 28/61 (45.9%) of OBI samples. Two HSLD samples had substitutions in the ‘a’ determinant (L127P and G145A), which were also found in OBI samples. In total, 27 HSLD samples (96.4%) carried PreS2 substitutions, which was somewhat higher than in the OBI HBsAgNx-negative group (76.5%, *p* = 0.0332) and higher than in the OBI HBsAgNx-positive group (63.0%, *p* = 0.0023). Nine HSLD samples had deletions or start codon mutations in PreS2.

Genotypes for sequenced samples in this study are summarized in Table 4. One sample in the HSLD group was a mixed infection with genotypes B and C. The majority of samples in the HSLD group was genotype E, reflecting the predominance of this genotype in the country of origin for these samples, Cote D’Ivoire [19].

### 3.4. PreS1, PreS2, and S Substitutions Unique to OBI or HSLD Samples

The OBI HBsAgNx-negative and HSLD sample groups in this study had similar low levels of HBV DNA, but very different levels of HBsAg. To investigate the possible role that substitutions in PreS1, PreS2, and S may play in these profiles, sequences from all three regions were analyzed to identify substitutions unique to the OBI or HSLD samples. Table 5 and Table 6 summarize the results of this analysis.

Unique PreS1 substitutions were found in both the OBI and HSLD groups (Table 5). Among OBI samples, a deletion at positions 57–99 was found in an HBsAgNx-negative sample, while unique deletions at amino acids 66–76 and 94 were observed in the HBsAgNx-positive group. Deletions at amino acids 7–29, 71–97, and 84–87 were unique to the HSLD group. Nine HSLD samples had a substitution at amino acid 85.

Multiple samples in the HSLD group had PreS2 substitutions at amino acid 6 or 39 or 52 that were not observed in the OBI population (Table 5). A deletion at amino acids 9–22 of PreS2 was observed in an HBsAgNx-negative OBI sample and deletions at positions 8–19 and 21–22 were observed in two HBsAgNx-positive OBI samples.

S substitutions unique to the OBI and HSLD sample groups are shown in Table 6. Substitutions at eight positions in the ‘a’ determinant of the MHR were observed in the OBI group that were not found in the HSLD sample group.

### 3.5. Detection of OBI and HSLD Samples by Novel Biomarker Assays

Representative samples from the OBI and HSLD sample groups were tested with two new biomarker assays, HBV pgRNA and HBcrAg, to assess the potential utility of these assays relative to sensitive HBsAg testing (Table 7). The mean HBV DNA level for the OBI samples shown in Table 7 was 29.7 IU/mL (range 1.26–363.1 IU/mL). Two of fourteen HBsAgNx-negative OBI samples were positive by both the pgRNA and HBcrAg assays. Only one of nine HBsAgNx-positive samples was detected by the HBcrAg assay, while none were detected by pgRNA. More than half of the HSLD group was not detected by pgRNA or HBcrAg, likely due to the low viral loads in these samples.

## 4. Discussion

The detection of early acute and occult HBV infections is a challenge for HBsAg immunoassays [4,5]. In the present study, of 319 NAT yield and OBI samples from five geographically diverse regions, the new HBsAg assay (ARCHITECT HBsAgNx) increased the detection of NAT yield samples by 33.6% and OBI samples by 22.3%; thus, allowing an improved diagnosis of these two clinically important groups of HBV-infected individuals. Mean HBV DNA concentrations were significantly lower in the HBsAgNx-negative sample groups (25.9–50.3 IU/mL) than in the HBsAgNx-positive sets (139.5–384.1 IU/mL), but comparable to the level (32.4 IU/mL) in the comparison HSLD group of 28 samples with readily detectable HBsAg (Table 1).

During most phases of HBV infection, HBsAg is produced in large excess over complete virions, but the ratios vary with the phase of infection. During the pre-ramp-up phase of early acute infection, viremia is low and HBsAg may be at too low a level to be detected. At the time of seroconversion to HBsAg positivity, excess HBsAg in subviral particles to HBsAg in the virus has been estimated at 100–1000-fold [20]. Based on the molecular weight of an HBsAg molecule of 25,000 Daltons and 240 HBsAg molecules per virion, the weight of HBsAg in one virion was calculated to be 0.00001 pg or 1 × 10^−8^ IU HBsAg [5]. Assuming an excess of HBsAg over the HBV DNA of 1000-fold for NAT yield samples, the HBsAgNx analytical sensitivity of 0.005 IU/mL could be expected to detect 500 particles/mL [5]. This corresponds to approximately 100 IU HBV DNA/mL [5,7]. Data for HBsAgNx-positive NAT yield samples shown in Table 1 and Figure 1 were consistent with this estimate. HBsAgNx detected 35/48 (73%) of samples with viral loads ≥100 IU/mL; all of the HBsAgNx-positive NAT yield samples had viral loads ≥38.0 IU/mL.

During later phases of infection, a 2700-fold excess of HBsAg has been reported for HBeAg-positive samples [5]. Assuming this ratio of excess HBsAg, the HBsAgNx analytical sensitivity of 0.005 IU/mL could be expected to detect 200 virus particles/mL [5]. This corresponds to approximately 40 IU HBV DNA/mL [5,7]. Data for HBsAgNx-positive OBI and HSLD samples indicated a much greater excess of HBsAg, possibly reflecting the later anti-HBe-positive phases of infection in these sample groups. Twenty-three OBI samples detected by HBsAgNx had viral loads between 1 and 10 IU/mL, which suggested HBsAg levels in excess of 10,000–100,000-fold over HBV DNA-containing virions. Five HSLD samples (genotype E) with very low viral loads between 1 and 10 IU/mL had HBsAg levels from 1268 to 12,740 IU/mL, more than a million-fold excess of HBsAg subviral particles over virions. Low amounts of HBV DNA with high HBsAg levels have been previously reported for genotype E samples [21]. It was hypothesized that the high levels of HBsAg were derived from S gene sequences integrated into the host genome. Not all samples with HBV DNA levels above 40 IU/mL were detected by HBsAgNx, prompting our investigation of factors beyond analytical sensitivity that may have contributed to the lack of detection.

The masking of HBsAg by anti-HBs has been proposed as one reason for the lack of the detection of OBI even if anti-HBs is undetectable [5]. In a previous report, we demonstrated that the new HBsAgNx assay had improved sensitivity for HBsAg even in the presence of high levels of anti-HBs [10,11]. In that study, HBsAg levels as low as 0.06 IU/mL were detectable by HBsAgNx in the presence of anti-HBs levels as high as 10,000 IU/mL, although S/CO values declined somewhat with increasing concentrations of anti-HBs [10]. Consistent with that observation, results from the present study showed that 20.9% of samples incrementally detected by HBsAgNx were anti-HBs-positive. However, data in Figure 2 suggest that anti-HBs levels over 300 mIU/mL may affect the detection of samples with extremely low viral loads (median viral load 4.42 IU/mL, corresponding to about 2.5 × 10^−7^ IU of virion bound HBsAg/mL). Detection of such samples would require at least a 20,000-fold excess of HBsAg in order to reach the detection limit of the HBsAgNx assay, with virtually no reduction in the signal by anti-HBs. If the excess level of HBsAg secretion relative to virions was below the 20,000-fold threshold, then the lack of detection could simply be due to HBsAg levels below the analytical sensitivity of the assay. The presence of anti-HBs in these samples may reflect the host humoral control of the infection leading to lower HBsAg levels.

In the present study, there was no significant difference between HBsAgNx-negative and HBsAgNx-positive NAT yield or OBI groups in the frequency of samples with substitutions or in the average number of substitutions per sample for the PreS1, PreS2, or S regions (Table 1). In contrast, there were significantly more S substitutions in the OBI samples than in the comparison HSLD group in which HBsAg was readily detectable (Table 1 and Table 6), a finding similar to other reports on the increased frequency of MHR substitutions in OBI [16,22,23,24,25]. HBsAg substitutions, particularly in the ‘a’ determinant within the MHR, have been associated with the lack of detection by affecting antigen binding to antibodies used in HBsAg immunoassays [16]. Previous studies have demonstrated that the HBsAgNx assay has an enhanced detection of substitutions in the MHR, including samples with insertions and single, double, or multiple substitutions [9,11,12]. Notably, two HSLD samples with readily detectable HBsAg had two ‘a’ determinant substitutions (L127P and G145A) that were also found in OBI samples.

The ‘a’ determinant substitutions observed in the HBsAgNx-negative samples in this study were shown to be detected by the HBsAgNx assay when the HBsAg level was at or above the assay detection limit, including samples with substitutions at C124, C139, and C147 involved in disulfide bonds of the ‘a’ determinant secondary structure [9,12]. Three OBI samples in the current study had substitutions at C124; all three were HBsAgNx-positive. Two HBsAgNx-negative OBI samples carried a substitution at C147 and one HBsAgNx-negative OBI sample had C139CY. C124Y, C139R, and C147R/A have been reported to impair virion or S protein secretion in HuH7 cells [16,26]. Thus, the observed HBsAgNx-negative results were likely due to very low levels of virus rather than assay failure. A similar conclusion was reported in a study comparing the HBsAg detection of six native samples carrying ten ‘a’ determinant mutations with recombinant proteins derived from the native samples [25]. Although the native samples were HBsAg-negative by sensitive immunoassays, all ten of the recombinant proteins were detected. The authors concluded that the lack of detection was the low level of HBsAg present in the native samples along with possible masking of HBsAg in immune complexes rather than mutations affecting immunoassay detection.

In the present study, eight OBI samples had MHR substitutions reported to impair the secretion of virions and subviral particles in HuH7 cells and mice, leading to reduced HBsAg levels below assay detection limits [16]. These substitutions (G119R, Q129R, T140I, and D144A) were not found in the HSLD group in the current study. A study by Ito et al. reported that the detection of G119 mutants depended on the antibodies and formats used in their detection methods rather than the mutation causing reduced HBsAg secretion [27]. However, Huang et al. reported that mice that received the G119R mutation showed an accumulation of HBV DNA and the retention of HBsAg in the liver [16]. Notably, some samples with the G119R and Q129R substitutions were detected by HBsAgNx (Table 3), including an anti-HBs-positive OBI sample with Q129R and two OBI samples with the G119R mutation amid multiple other substitutions within the MHR. In addition, recombinant S proteins with G119 and Q129 mutations have been shown to be detected by HBsAgNx when ≥0.005 IU/mL [9]. These results emphasize that HBsAg assay sensitivity and the robust detection of variants continue to be critical parameters of assay performance.

Reductions in HBsAg synthesis and secretion may also be mediated by substitutions and deletions in the PreS1 and PreS2 regions and nonsense point mutations in the S region [28,29,30,31,32]. PreS1 deletions can block the transcription of the 2.1 kb RNA for HBsAg [31]. In the present study, PreS1 and PreS2 deletions at six locations were observed in OBI samples that were not present in the HSLD group (Table 5) which may have led to the low or undetectable levels of HBsAg in these samples. For example, in one HBsAgNx-negative OBI sample (genotype A1), PreS1 amino acids 57–99 were deleted (Table 5). The region of the deletion overlapped with the SPII promoter for transcription of the 2.1 kb RNA. Similar deletions within the SPII promoter region have been reported to result in the loss of HBsAg production in HuH7 cells [31]. One HBsAgNx-positive OBI sample had the sW182* mutation associated with a reduced HBsAg secretion [32].

Interestingly, eight of twenty genotype E HSLD samples also had deletions or start codon mutations in PreS1/PreS2, yet had readily detectable levels of HBsAg. Two HSLD samples had the sL216* mutation which was associated with low or undetectable HBsAg in transfection studies [33]. These anomalous results might be explained by a high rate of S gene integration in the host genome that has been suggested to occur in genotype E, as well as the compartmentalization of viral strains circulating in plasma from those in the liver [21].

Host factors must also be considered in addition to immunoassay and viral causes for the lack of HBsAg detection. In most OBI cases, virus replication and transcription are strongly suppressed by host defense mechanisms (both T cell and humoral) [29,34,35]. Bes et al. reported that the increased T-cell response in OBI led to the reduction in HBsAg expression to undetectable levels and that the lack of HBsAg detection was due to low production rather than mutations or immunoassay failure [24]. In the current study, the OBI sample group had a higher rate of anti-HBs positivity than the HSLD comparison group (41.5% vs. 3.6%), and within the OBI group anti-HBs was more prevalent in the HBsAgNx-negative samples than among the HBsAgNx positives (45.2% vs. 28.6%). Recent reports have indicated that anti-HBs may inhibit the secretion of subviral particles [36]. Whether varying degrees of immune control might lead to a continuum of HBsAg levels below the detection limits of even highly sensitive HBsAg assays is a topic for further study.

Finally, we investigated whether two new HBV biomarker assays, HBV pgRNA and HBcrAg, would be of value in improving the detection of OBI samples. The level of pgRNA in samples from untreated chronic HBV patients is typically 2.2 log U/mL lower than the HBV DNA level [13]. Based on the low HBV DNA levels observed in OBI samples, a low rate of detection by the pgRNA assay might be expected. Indeed, in the current study, using a subset of the OBI sample group with a mean HBV DNA level of 29.7 IU/mL (range 1.26–363.1 IU/mL), only two of twenty-three OBI samples were pgRNA-positive. HBcrAg has been shown to correlate with HBV DNA levels and has been reported to be undetectable or at very low levels in most HBeAg-negative inactive carriers, suggestive of low cccDNA levels leading to low viral replication [37]. In the current study, HBcrAg was below the assay measuring range in 20 of 23 OBI samples with low level HBV DNA (mean 29.7 IU/mL) and near the HBcrAg measuring range lower limit in two samples. Thus, the results of this limited study were consistent with expected results based on sample viral loads and indicated that neither pgRNA or HBcrAg provided a significant incremental detection of untreated OBI compared to the HBsAgNx assay.

The current study was limited to the sequencing and analysis of the PreS1, PreS2, and S gene regions. Mutations in viral regulatory elements or combinations of mutations throughout the genome may also be involved in the control of HBsAg synthesis and secretion [29].

In conclusion, there is a need for more sensitive HBsAg assays to improve the detection of early acute and occult hepatitis B infections [5,35,38]. The clinical implications of these infections include the potential for HBV transmission and HBV reactivation in OBI cases undergoing potent immunosuppressive therapies [35]. This study demonstrated that the new HBsAgNx assay provided an increased detection of both NAT yield (33.6%) and OBI (22.3%) samples from five geographically diverse regions. Nevertheless, there were some NAT yield and OBI samples in which HBsAg was undetectable. That fact led us to investigate viral, host, and immunoassay factors that might underly HBsAg assay negativity in these samples. The evidence indicated that the lack of HBsAgNx detection in these sample groups was likely due to exceptionally low HBsAg levels below the detection limit of the assay. Additional studies are needed to determine if further technical advances such as digital immunoassays can provide the sensitivity and specificity required for the detection of significantly more infections in a practical and cost-effective way.

## Figures and Tables

**Table 1:** Features of Sample Groups Evaluated in this Study (Total of 347 Samples).

	Sample Group				
	HBV NAT Yield HBsAg Qual II(−), HBV DNA(+), Anti-HBc(−) N = 131		Occult HBV Infection (OBI) HBsAg Qual II(−), HBV DNA(+), Anti-HBc(+), N = 188		HSLD (High HBsAg with Low HBV DNA Levels) Anti-HBc(+) N = 28
HBsAg (IU/mL)	<0.005 ^a^ HBsAgNx(−)	0.005–0.02 ^b^ HBsAgNx(+)	<0.005 ^a^ HBsAgNx(−)	0.005–0.02 ^b^ HBsAgNx(+)	Mean = 10,104 ^c^ Median = 6050.5 ^c^
N Samples (% of total)	87 (66.4)	44 (33.6)	146 (77.7)	42 (22.3)	28 (100)
HBV DNA					
Mean IU/mL	50.3	384.1	25.9	139.5	32.4
Range	1.2–676.1	38.0–1995.0	1.1–363.1	1.3–1995.0	2.0–85.0
	*p* < 0.0001		*p* = 0.0005		
Anti-HBs					
N ≥ 10 mIU/mL (%)	36 (41.4)	6 (13.6)	66 (45.2)	12 (28.6)	1 (3.6)
	*p* = 0.0014		*p* = 0.0748		
N sequenced	45	43	34	27	28
Genotypes	A1,A2,B,C,E,F,H	A1,A2,B,C,E	A1,A2,B,C,D,E	A1,A2,A7,B,C,D,E	B,C,E
N Samples with PreS1 Substitutions	32 (71.1%)	23 (53.5%)	30 (88.2%)	24 (89.9%)	26 (92.9%)
Mean Substitutions (range)	1.50 (1–3)	1.70 (1–4)	2.37 (1–6)	1.75 (1–5)	3.77 * (1–12)
N Samples with PreS2 Substitutions	34 (75.6%)	30 (69.8%)	26 (76.5%)	17 (63.0%)	27 ** (96.4%)
Mean Substitutions (range)	1.94 (1–4)	2.10 (1–5)	3.0 (1–8)	2.65 (1–6)	3.0 (1–8)
N Samples with S Substitutions	25 (55.6%)	21 (48.8%)	27 (79.4%)	21 (77.8%)	24 (85.7%)
Mean Substitutions (range)	1.24 (1–3)	1.52 (1–5)	7.44 (1–26)	7.10 (1–18)	3.46 *** (1–9)
N Samples with S Escape Mutation	2 (4.4%)	1 (2.3%)	16 (47.1%)	12 (44.4%)	2 (7.1%)
N Samples with Substitutions in All Three Regions	17 (37.8%)	11 (25.6%)	22 (64.7%)	13 (48.1%)	24 (85.7%)

^a^ ARCHITECT HBsAg Qualitative II (HBsAg Qual II)(−) and ARCHITECT HBsAg NEXT (HBsAgNx)(−). HBV surface antigen (HBsAg) level was based on the limit of detection of ARCHITECT HBsAgNx. ^b^ ARCHITECT HBsAg Qualitative II(−) and ARCHITECT HBsAgNx(+). HBsAg level was based on limits of detection for ARCHITECT HBsAg Qualitative II and HBsAgNx. ^c^ Tested with quantitative ARCHITECT HBsAg assay. * *p* = 0.0191 compared to OBI HBsAgNx(−), *p* = 0.0015 compared to OBI HBsAgNx(+). ** Nine samples had deletions or start codon mutations in PreS2. *** *p* = 0.0080 compared to OBI HBsAgNx(−), *p* = 0.0059 compared to OBI HBsAgNx(+). N indicates number of samples. NAT (nucleic acid test), anti-HBs (antibody to HBsAg).

**Table 2:** Data for NAT yield samples with substitutions in amino acids 100-160 of the HBsAg S protein.

Sample	Anti-HBs	HBsAgNx	Genotype	S Protein Amino Acid Substitutions
SANBS83	−	−	A1	T143M
254	−	−	C2	Q101H, R160S
AM8.98000	−	−	B4	T131N
BM7.00531	−	−	B4	P127T
BM6.33816	−	−	C1	Y100C
264	−	+	E	I110IL
97	−	+	A2	Y100S
291	−	+	A1	A159G
292	−	+	C5	L110I, R160K
AM9.92991	−	+	B4	A128V
BM9.02298	−	+	B4	I110L, T143M
BM3.32643	−	+	B4	I110L, S113T
300	+	−	H	S143T

Anti-HBs values ≥10 mIU/mL are shown as positive(+). Anti-HBs values <10 mIU/mL are shown as negative(−). HBsAg NEXT assay results are indicated as negative(−) or positive(+).

**Table 3:** Data for OBI samples with substitutions in amino acids 100-160 of the HBsAg S protein.

Sample	Anti-HBs	HBsAgNx	Genotype	S Protein Amino Acid Substitutions
HBV0150	−	−	A2	Y100C, Q101R, M103I, I110F, S113P, S117G, T123P, T126I, G130GD, N131K, F134V, T143S, C147Y
102	−	−	A2	M103I
ARC77	−	−	A2	Q101R, G145A
AM7.98526	−	−	B4	M133L
BM5.15420	−	−	B4	M133L
BM8.11637	−	−	B4	T123N, F134C
BM3.26184	−	−	C1	S154P
270	−	+	E	I110IL
271	−	+	E	I110L, C124Y
274	−	+	E	Q101R, L127P, D144E
HBV0316	−	+	D3	G119R, P120L, R122Q, C124S, M133I, T140S, K141N, P142L, D144G
ARC44	−	+	A1	V106I
ARC09	−	+	B2	L109M, M133L
ARC48	−	+	B4	L109M, M133L, F134V
ARC82	−	+	D3	G112N, A128V, T131A, M133I
BM2.11547	−	+	B4	Y100W, Q101QL, Q129H
BM7.00672	−	+	B4	S114A, G119R, P120Q, C124S, T126I, Q129P, G130K, T131N, S132F, M133T
BM9.06187	−	+	C1	Y100C, G145A
SANBS26	+	−	A1	Q101H, F134V, P142L, D144A
SANBS63	+	−	A1	N131H
SANBS96	+	−	A1	G112E, T118M, P120PT, M133T, T140I
HBV0003	+	−	A1	Q101QR, M103V, S117SN, T118TM, P120PS, A128V, M133MT, C139CY, D144E
OBI7096	+	−	D1	Q101L, S117T, P120S, M133T, Y134F, S136Y, D144A, S154P
OBI7063	+	−	D2	Y100C, T118A, P120Q, P127T, Q129R, S136Y, K160N
OBI7086	+	−	D3	Q101QR, G112R, S113T, T123A, P127I, A128V, G130GR, P142PL, D144E, C147CY
ARC50	+	−	A2	122Ins 2aa
ARC56	+	−	A2	Y100C, Q101QC, M103I, N131K, M133I, D144E, S154P
AM8.89075	+	−	B4	Q101R, L104F, K141R, D144A, K160N
BM8.05932	+	−	B4	P120S, G130GR, T131TN, M133T
BM4.15392	+	−	B4	Y100F, Q101R, L109R, I110L, S113T, P120A, K122T, P127L, F134S, D144E, K160V
BM3.32210	+	−	B4	Y100YC, Q101R
279	+	+	A7	Y100C
262	+	+	E	I110IL
ARC79	+	+	A2	D144N
BM2.27608	+	+	B4	P127A, Q129R, T131N, M133S, F134Y
AM9.94494	+	+	B4	A159GV

Anti-HBs values ≥10 mIU/mL are shown as positive(+). Anti-HBs values <10 mIU/mL are shown as negative(−). HBsAg NEXT assay results are indicated as negative(−) or positive(+).

**Table 4:** Genotype results for sequenced samples.

Genotype	NAT Yield and OBI		N High HBsAg–Low DNA (HSLD) (HBsAg ≥ 0.05 IU/mL)
	N HBsAgNx(−) (HBsAg < 0.005 IU/mL)	N HBsAgNx(+) Qual II (−) (HBsAg 0.005–0.02 IU/mL)	
A1	12	14	0
A2/A7	13	15	0
B	33	17	5 *
C	12	10	3
D	4	2	0
E	3	12	20
F1	1	0	0
H	1	0	0
Total N sequenced	79	70	28

149 NAT yield and OBI samples were sequenced: 79 representing the HBsAg NEXT-negative group and 70 representing the HBsAg NEXT-positive group. All 28 samples in the HSLD sample group were sequenced. * One sample was a mixed infection with genotypes B and C. N indicates number of samples.

**Table 5:** PreS1 and PreS2 amino acid substitutions and deletions unique to OBI or HSLD samples.

Sample Group	PreS1	PreS2
OBI HBsAgNx(−)	F25L(2), A28T, K57T, del 57-99, P65L, S78N, P89T, N98NK, N98NT, N98I, G102R	del 9-22, A11T(2), P36Q, P54Q
OBI HBsAgNx(+)	S5L, H50Q(3), del 66-76, del 94	del 8-19, L20K, del 21-22, A24V, S28N, N55T
HSLD	del 7-29, H15Q, T18S(2), D27G(3), R34K(2), R38K, D49N, A62S(3), F62C, F67L, T67I, T67N, T68I, H70V, del 71-97, G72S(3), G73S, G73N(3), W76S(2), S77I, Q79L, del 84-87, K85N, K85Q(4), K85T(4), T96S, Q99R, Q99K, S101V, G101R, I107L(2), D113E, D114E(3), A119V	T6P, T6K, T6S(5), del 8-22, F8Y, del 19-22, V39A(5), A39V, S43L, T49I, P52L(4), P52H(2)

Sequence data from 61 OBI and 28 HSLD samples were analyzed relative to the respective genotype consensus sequence. Substitutions and deletions (del) unique to each sample group are shown (i.e., the substitution or deletion was not found in either of the other two sample groups.) For a substitution found in more than one sample, the number of samples is indicated in parenthesis. When mixtures of substitutions and wild-type amino acids occurred, results are shown if the substitution comprised ≥50% of amino acids. HBsAg NEXT assay results for OBI samples are indicated as negative (−) or positive (+).

**Table 6:** S amino acid substitutions and deletions unique to OBI or HSLD samples.

Sample Group	S Amino Acids 1–99	S Amino Acids 100–226
OBI HBsAgNx(−)	E2G(2), F19Y, T27A, P29PL, S31N, P62L, P62F, I92T, F93L, F93C(2), L94S, L97H, L98R	M103I(3), M103V, L104F, S113P, S113T(2), T118M, T118TM, T118A, K122ins., T123P, T123N, T123A, **S136Y(2), C139CY, T140I, T143S, D144A(3), C147Y, C147CY**, S167L, G185GE, F219S, C221Y
OBI HBsAgNx(+)	S6SL, S6T, T23NI, I28T, T45K, S55Y, I57IT, L84LF, I86T, L95S	G119R(2), **C124Y, C124S(2), S132F**, W182*, P188PL, S193L, M197MT
HSLD	L13R, A17E(2), L21S(2), L21W, S58L(5), R73H, M75T, F80S	T115N, T116N, T189I(2), F200Y(4), L216*(2), I218L

Sequence data from 61 OBI and 28 HSLD samples were analyzed relative to the respective genotype consensus sequence. Substitutions, deletions, and insertions (ins.) unique to each sample group are shown (i.e., the substitution or deletion was not found in either of the other two sample groups). For a substitution found in more than one sample, the number of samples is indicated in parenthesis. When mixtures of substitutions and wild-type amino acids occurred, results are shown if the substitution comprised ≥50% of amino acids. One OBI HBsAgNx(−) sample had a two amino acid insertion at amino acid position 122 of the S protein. Amino acid substitutions in the ‘a’ determinant are indicated in bold. HBsAg NEXT assay results for OBI samples are indicated as negative (−) or positive (+).

**Table 7:** Detection of representative OBI and HSLD samples by novel biomarker assays.

	Sample Group		
	OBI		HSLD
HBsAg (IU/mL)	<0.005 ^a^	0.005–0.02 ^b^	≥0.05 ^c^
HBV Pregenomic RNA (pgRNA)			
N detected/N tested	2 */14 (14.3%)	0/9 (0%)	12/26 (46.2%)
Log U/mL for detected samples	<1.65, 4.09		All <1.65 ^d^
HBV core-related antigen (HBcrAg)			
N detected/N tested	2 */14 (14.3%)	1/9 (11.1%)	12/28 (42.9%)
Log U/mL for detected samples	3.1, ≥6.8	3.0	Mean = 3.5 **

^a^ ARCHITECT HBsAg Qualitative II(−) and ARCHITECT HBsAgNx(−). HBsAg level is based on the limit of detection of ARCHITECT HBsAgNx. ^b^ ARCHITECT HBsAg Qualitative II(−) and ARCHITECT HBsAgNx(+.) HBsAg level is based on limits of detection for ARCHITECT HBsAg Qualitative II and HBsAgNx. ^c^ HBsAg levels determined by quantitative ARCHITECT HBsAg assay. ^d^ Twelve HSLD samples were pgRNA-positive but below the quantitation range of the pgRNA assay. * Same two samples. N indicates number of samples. ** Range 3.1–4.3 log U/mL.

## Data Availability

Data are contained in this article or available on request.
